# Genome-wide identification and characterization of long intergenic noncoding RNAs and their potential association with larval development in the Pacific oyster

**DOI:** 10.1038/srep20796

**Published:** 2016-02-10

**Authors:** Hong Yu, Xuelin Zhao, Qi Li

**Affiliations:** 1 Key Laboratory of Mariculture, Ministry of Education, Ocean University of China, Qingdao 266003, China

## Abstract

An increasing amount of evidence suggests that long intergenic noncoding RNAs (lincRNAs) may play diverse roles in many cellular processes. However, little is known about lincRNAs in marine invertebrates. Here, we presented the first identification and characterization of lincRNAs in the Pacific oyster (*Crassostrea gigas*). We developed a pipeline and identified 11,668 lincRNAs in *C. gigas* based on RNA-Seq resources available. These lincRNAs exhibited many common characteristics with vertebrate lincRNAs: relatively short length, low exon numbers, low expression, and low sequence conservation. 1,175 lincRNAs were expressed in a tissue-specific manner, with 35.2% preferentially expressed in male gonad. 776 lincRNAs were specifically expressed in juvenile during different developmental stages. In addition, 47 lincRNAs were found to be potentially related to oyster settlement and metamorphosis. Such diverse temporal and spatial patterns of expression suggest that these lincRNAs might function in cell differentiation during early development, as well as sex differentiation and reproduction. Based on a co-expression network analysis, five lincRNAs were detected that have an expression correlation with key hub genes in four modules significantly correlated with larval development. Our study provides the first large-scale identification of lincRNAs in molluscs and offers new insights into potential functions of lincRNAs in marine invertebrates.

Over the past decade, with the development of next-generation sequencing techniques, genome-wide transcriptome analysis discovered that the genomes of eukaryotes encode a large number of noncoding transcripts^1^,^2^,^3^. In mammals, approximately two thirds of transcribed loci do not encode protein, many of which are intergenic and long (>200 bp) noncoding RNAs (lncRNAs)^4^,^5^. Long intergenic noncoding RNAs (lincRNAs, also called long intervening noncoding RNAs) are a recently defined class of noncoding RNAs derived from the intergenic regions of the genome^6^, which occur in all eukaryotic model organisms that have been investigated up to now^7^,^8^,^9^,^10^. Although the majority of lincRNAs express at lower levels compared with protein-coding mRNAs and lack protein coding potential, emerging evidence indicates that lincRNAs have widespread roles in many cellular processes, including gene regulation, dosage compensation, apoptosis, embryonic development, imprinting, cellular differentiation, and etc^1^,^11^,^12^,^13^,^14^. More and more lincRNAs have been validated and shown to possess functions, as opposed to being transcriptional noise. However, the mechanism underlying how lincRNAs function is as yet largely unknown.

To date, only a small portion of lincRNAs in some model organisms have been characterized experimentally in detail^11^,^12^,^13^. In contrast to mRNAs, the sequence of lincRNAs has a high evolution rate^6^, making it difficult to identify and predict its function solely based on the sequences. More recently, with the aid of next-generation sequencing techniques and new computational analysis, it has facilitated the identification and characterization of lincRNAs in many species. Meanwhile, identification of lincRNAs has been continuously reported in many species, including both animals and plants (eg. mammals, chickens, flies, zebrafish, *Arabidopsis*, cucumber and maize)^1^,^2^,^7^,^8^,^13^,^15^,^16^,^17^. However, the systematic identification of lincRNAs in marine invertebrates has received little attention, although marine invertebrates are rich in species.

The Pacific oyster, *Crassostrea gigas*, is a widely distributed and commercially important species, belonging to Mollusca, which contains the largest number of described marine animal species^18^. With a typical molluscan larval ontogeny, the developmental process of *C. gigas* comprises of three principal stages: trochophore, veliger and metamorphosis into the juvenile^19^. *C. gigas* is a unique model for developmental studies because of its dramatic changes during larval development. Owing to its economical, biological and ecological importance, the biology and genetics of the Pacific oyster have been extensively studied^20^,^21^,^22^,^23^, which enables *C. gigas* to be a potential model organism for marine molluscan studies. However, the characteristics and function of lincRNAs of *C. gigas* are, as yet, largely unknown. The recently released genome sequence of *C. gigas* provides an excellent reference for genome-wide exploration of lincRNAs in this species^19^. Moreover, hundreds of transcriptome data for *C. gigas* are now publically accessible^19^, which enables the systematic identification and characterization of oyster lincRNAs.

In this study, we carried out the first genome-wide scale catalog of lincRNAs in *C. gigas* using transcriptome data generated by RNA-Seq. Tissue- and developmental stage-specific expression profiles were analyzed to determine the expression patterns of oyster lincRNAs. The potential function of lincRNAs in larval development of oysters was investigated based on lincRNA-mRNA co-expression networks. Our study not only enrich the knowledge of noncoding RNAs in marine invertebrates, but also provide new insights into potential functions of lincRNAs in molluscs.

## Results and Discussion

### Identification and characterization of oyster lincRNAs based on RNA-Seq datasets

**Figure 1:**
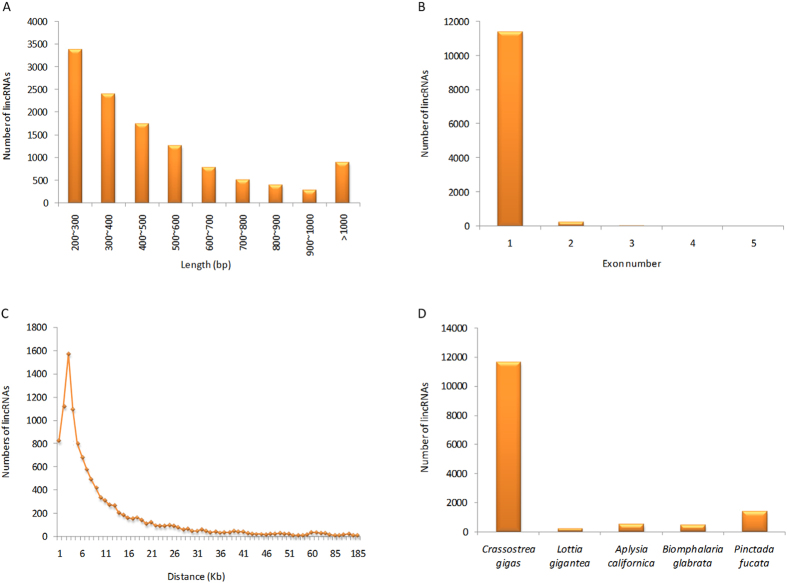
Characteristics of lincRNAs in the Pacific oyster. (**A**) Length distribution of 11,668 lincRNAs. (**B**) Distribution of exon numbers in lincRNAs. (**C**) The nearest distance between lincRNAs and their neighboring protein-coding genes. (**D**) The conservation of oyster lincRNAs in four molluscs.

In order to identify a relatively comprehensive set of *C. gigas* lincRNAs, 45 RNA-Seq datasets from different tissues and developmental stages were selected (Table S1), and subsequently subjected to a stringent filtering pipeline. In total, our pipeline yielded 11,668 potential lincRNAs with their genomics position provided in Table S2. The length of the lincRNAs ranged from 200 to 5,891 bp, of which lincRNAs of 200–300 bp were the most abundant (28.9%, Fig. 1A). The majority of the lincRNAs were relatively short with a few (7.7%) greater than 1 kb in length. The average length was 508 bp, which was shorter than that of the protein-coding genes (average length = 1,317 bp). This feature was similar to that observed in vertebrates^1^,^13^,^24^. Most (97.7%) of the lincRNAs in *C. gigas* contained only a single exon (Fig. 1B). We found that 68.0% of the total lincRNAs was transcribed within in a region less than 10 kb from the protein-coding genes (Fig. 1C), suggesting that many lincRNAs were byproducts of mRNA biogenesis^25^. Mammalian and zebrafish lincRNAs have also been reported to tend to be within <10 kb of protein-coding genes^6^,^10^.

### Conservation of oyster lincRNAs

To determine the sequence conservation of oyster lincRNAs in Mollusca, we searched the 11,668 oyster lincRNA sequences against the genome sequences of other four molluscs (*Lottia gigantea, Aplysia californica, Biomphalaria glabrata, Pinctada fucata*) using BLASTN. The results indicated that 211–1,381 oyster lincRNAs were conserved compared to the other four molluscs (Fig. 1D), and 46 oyster lincRNAs had sequence homology in all four molluscs. Previous studies indicate that a high rate of sequence evolution is a general feature of animal lincRNAs. In vertebrates, it had been reported that lincRNA sequences evolved very rapidly^6^,^10^. Less than 6% of zebrafish lincRNAs had orthologs in human or mouse^10^. Consistent with the data from vertebrates, in this study, we also found low sequence conservation of lincRNAs in molluscs (1.8–11.8%). However, the rate of lincRNAs might be overestimated in the present data because the conservation of lincRNA sequence was evaluated based on whole-genome alignments, which compared the whole genome sequences rather than lincRNA sequences in other molluscs. The presence of a segment homologous to an oyster lincRNA in other molluscan genomes did not definitively prove that the homologous segment was part of a lincRNA in other molluscs. Therefore, the lincRNA sequences in molluscs might be even more poorly conserved than those in vertebrates.

### Tissue expression profile of oyster lincRNAs

One of the most striking features of lincRNAs is their extremely tissue-specific expression^9^, which may be key to their function^26^. In this study, the tissue-specific expression of lincRNAs was investigated using RNA-Seq datasets from nine tissue types: gill, hemolymph, digestive gland, labial palp, female gonad, male gonad, inner part of mantle, outer edge of mantle and adductor muscle.

**Figure 2:**
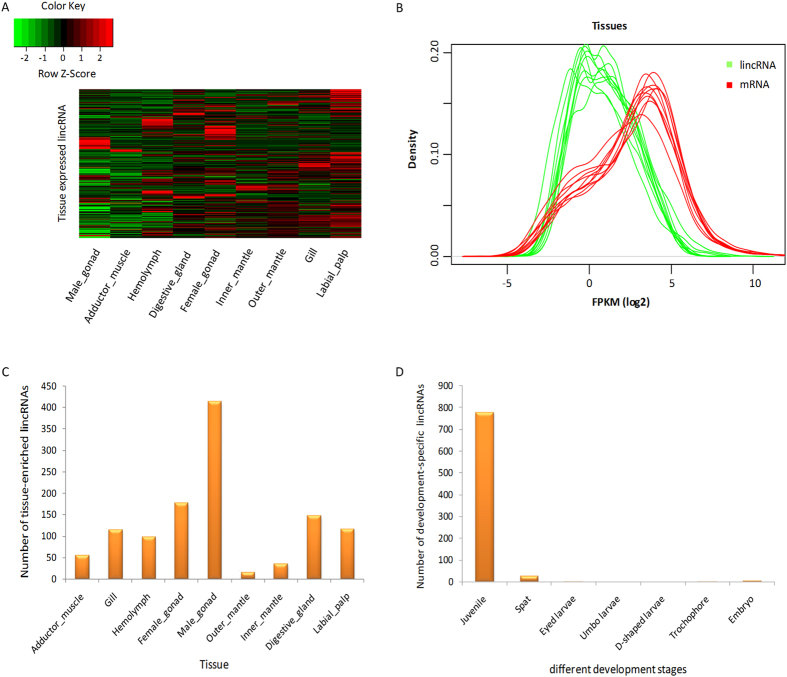
Expression patterns of lincRNAs in the Pacific oyster. (**A**) Heat map of lincRNAs expressed in nine tissues. Columns represent tissues, and rows represent the 8,489 lincRNAs expressed in tissues. Green indicates low expression; red, high expression. (**B**) Different expression levels of lincRNAs and mRNAs in each tissue dataset. The red and green curves represent mRNAs and lincRNAs, respectively. (**C**) The number of tissue-enriched lincRNAs in the nine tissue types. (**D**) The number of development-specific lincRNAs in seven developmental stages.

In total, we detected 8,489 lincRNAs expressed at least in one of the nine tissue types (FPKM ≥ 1), of which 46.4% had an expression of >5 FPKM in at least one tissue, suggesting that the lincRNAs serve a biological purpose, rather than simply representing transcriptional “noise”^16^ (Fig. 2A). The expression levels of lincRNAs and mRNAs were compared by Kernel density estimates (KDE). The results showed that the density peaks of protein-coding genes lagged behind those of lincRNAs in each dataset (Fig. 2B). The expression levels of lincRNAs were significantly lower than that of protein-coding genes (Kolmogorov-Smirnov test, *P* < 2.2e-16), which was also observed in many mammalian species and plants^8^,^9^,^16^,^24^.

Of the 8,489 lincRNAs expressed in various tissues, 5,800 (68.3%) were transcribed within 10 kb of the protein-coding neighbor genes. A total of 3,919 nearest protein-coding neighbors were collected for GO enrichment analysis. These nearest neighbors of tissue-specifically expressed lincRNAs were significantly enriched (*P* < 0.05) for 141 GO terms (Table S3), mainly referring to biological processes (cellular process, and biological regulation), molecular function (binding, and transferase activity), and cellular component (membrane, and cell junction). In vertebrates, the closest neighbors of lincRNAs were enriched for GO terms associated with transcription regulation^1^,^10^,^24^. The neighbor genes of *C. gigas* tissue-expressed lincRNAs are also likely to function in transcription-related processes (Table S3). It was reported that the lincRNAs, transcribed in close proximity to protein-coding genes, were likely to *cis*-regulate their neighbors, perhaps through transcriptional interference^27^. The tissue-expressed lincRNAs that were transcribed nearby (<10 kb) the protein-coding genes in *C. gigas*, perhaps, represented the best candidates for investigating the transcriptional regulation of neighbor genes.

The tissue-specific expression of lincRNAs was characterized using the tissue-specific index^28^, and the lincRNAs with tissue-specific index >0.95 were considered as tissue-specific lincRNAs. In total, 1,175 lincRNAs (13.8%) presented enriched expression in a single tissue type. Male gonad had the largest number (414) of tissue-enriched lincRNAs (Fig. 2C), suggesting that lincRNAs might have important roles in sex differentiation or male gonadogenesis in *C. gigas*. In agreement with our data, testis-specificity of lincRNAs was observed in many mammals, which suggested a role of lincRNAs in sexual selection or testis-specific process^6^,^9^,^29^. However, little is known about how the lincRNAs work in testis. Extensive investigation on the mechanisms of how lincRNAs function is needed.

### Temporal expression of lincRNAs at different developmental stages

The expression dynamics of lincRNAs were explored using RNA-Seq datasets obtained from 35 time-points of different developmental stages, including embryo stage, planktonic larval stages (trochophore, D-shaped larvae, umbo larvae, and eyed larvae), spat and juvenile stage (Table S1). A total of 10,685 lincRNAs were detected in the 35 time-points (FPKM ≥ 1), of which 809 lincRNAs showed development-specific expression (expressed specifically in one development stage). The distribution of the development-specific lincRNAs was extremely uneven in the seven development stages analyzed (Fig. 2D). As indicated, 776 lincRNAs were specifically expressed in juvenile, while six lincRNAs were specifically expressed in embryos, planktonic larvae and spat stages, but not in the juvenile. *C. gigas* has two distinct lifestyles (planktonic or sessile). According to the stage-specific expression of lincRNAs, we can infer that some lincRNAs induced in juvenile with sessile lifestyle may function in tissue differentiation, organogenesis, sex differentiation, and etc. The six lincRNAs that are specifically expressed in the larval stage might have potential roles in the free movement of larvae. Larval settlement and metamorphosis are important steps in the life cycle of sessile marine invertebrates, which accompany drastic changes of both their morphologies and lifestyles^30^. After metamorphosis, free-swimming larvae complete their planktonic lives and start benthic lives by transforming their body plan to the adult form^30^. Regulation of settlement and metamorphosis is critical for *C. gigas*, wherein the complicated regulatory mechanisms are as yet elusive. In this study, changes in the expression of lincRNAs between eyed larvae (competent pediveliger for metamorphosis) and spat (just after settlement and metamorphosis) were compared to investigate lincRNAs related to settlement and metamorphosis. We found that 21 lincRNAs showed moderate and high expression levels in eyed larvae, but not expressed in spat. In contrast, 26 lincRNAs only expressed in spat, but not expressed in eyed larvae. These results suggested that these 47 lincRNAs are candidates for functioning in oyster settlement and metamorphosis. In vertebrates, several studies have indicated that lincRNAs had diverse roles in vertebrate development^1^,^10^,^13^. For instance, Zhao *et al.* detected that lincRNAs were involved in fetal porcine skeletal muscle development^1^. In zebrafish, lincRNA *cyrano* has proved to be required for proper embryonic development and lincRNA *megamind* regulated brain morphogenesis and eye development^10^. However, we did not find any ortholog of the lincRNAs (*cyrano* and *megamind*) in the genome of *C. gigas*. Actually, among all lincRNAs detected in zebrafish, only three lincRNAs (Zv9_00057321, Zv9_00046280 and Zv9_00056161) had sequence homology in the genome of *C. gigas*, suggesting the rapid evolution of lincRNA sequences again.

### lincRNA-mRNA co-expression network during the larval development of oyster

**Figure 3:**
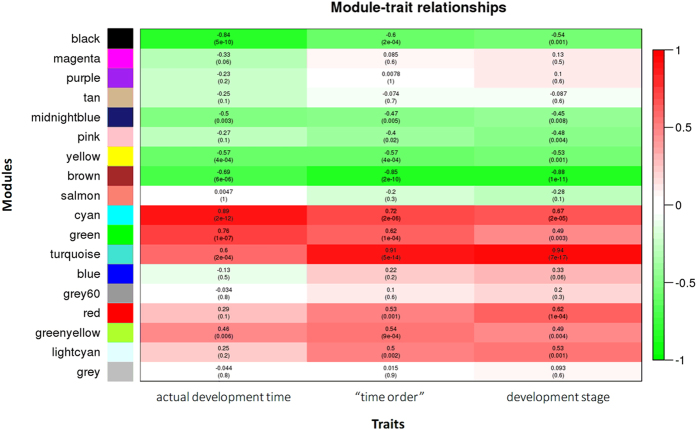
Module-trait relationships plot. Each row corresponds to a module, column to a trait. Each cell contains the corresponding correlation and *P*-value given in parentheses. The cells are color-coded by the correlation according to the color legend on the right, with red indicating strong positive correlation and green strong negative correlation.

To infer the potential function of lincRNAs in oyster larval development, a co-expression network between lincRNAs and mRNAs was constructed using the expression data and trait data. We tested three trait data: real time-points, “time order”, and development stages. Since the relationship between development time and gene expression was often non-linear, we sorted the development time and gave the lowest rank (1) to the shortest development time and the highest rank (34) to the longest development time. The “time order” data could be more suitable for the nonparametric correlation analysis. In addition, according to the development stages, we categorized the 34 samples into six development stages including embryo stage, trochophore stage, D-shaped larvae stage, umbo larvae stage, pediveliger stage and spat stage. The six development stages were also used as a trait dataset. A weighted gene co-expression network was developed to identify clusters (referred to as modules) of co-expressed genes (mRNAs or lincRNAs) for larval development. A total of 18 co-expression modules of densely interconnected genes were identified (Fig. 3), with the number of genes (both protein-coding genes and lincRNAs) in the modules ranging from 46 (grey60) to 12474 turquoise. The module-trait correlation analysis revealed that five modules were highly (Spearman correlation coefficient ≥0.6) and significantly (*P* < 0.01) correlated with larval development time, of which three modules (cyan, green and turquoise module) were positively correlated and two (black and brown module) were negatively correlated (Fig. 3). The number of mRNAs in the five modules varied from 71 (cyan) to 10,253 (turquoise), and the number of lincRNAs varied from eight (cyan) to 2,221 (turquoise) (Table S4). In the black module, 24 lincRNAs were negatively correlated with the development time (FDR-adjusted *P* < 0.05), suggesting that these lincRNAs were down-regulated with larval development. For the brown module, 353 lincRNAs had a negative relationship with the development time, while 106 lincRNAs had a positive correlation (FDR-adjusted *P* < 0.05). Two lincRNAs were detected positively correlated with the development time (FDR-adjusted *P* < 0.05) in the cyan module. In the green module, 51 lincRNA were positively correlated and 39 were negatively correlated (FDR-adjusted *P* < 0.05) with the development time. A total of 1,882 lincRNAs were detected with significant correlation with the development time in the turquoise module (FDR-adjusted *P* < 0.05), of which most (1,443) were positively correlated.

We performed the GO enrichment analysis for the five development trait-related modules and detected a significant enrichment (*P* < 0.05) of 30–160 GO terms in the five modules (Table S5). For example, in the two negatively correlated modules (black and brown), the enrichment of GO terms was found in several cellular processes including “negative regulation of apoptotic process”, “negative regulation of cell death”, “negative regulation of cellular macromolecule biosynthetic process”, “regulation of anatomical structure morphogenesis”, “regulation of multicellular organismal development”, “cell differentiation” and “anatomical structure development”. In the three positively correlated modules (cyan, green and turquoise), we found a significant enrichment in “ion binding”, “anion transport”, “ion homeostasis”, “catalytic activity”, “hydrolase activity”, “regulation of cellular component size”, and “regulation of cellular component biogenesis”.

**Figure 4:**
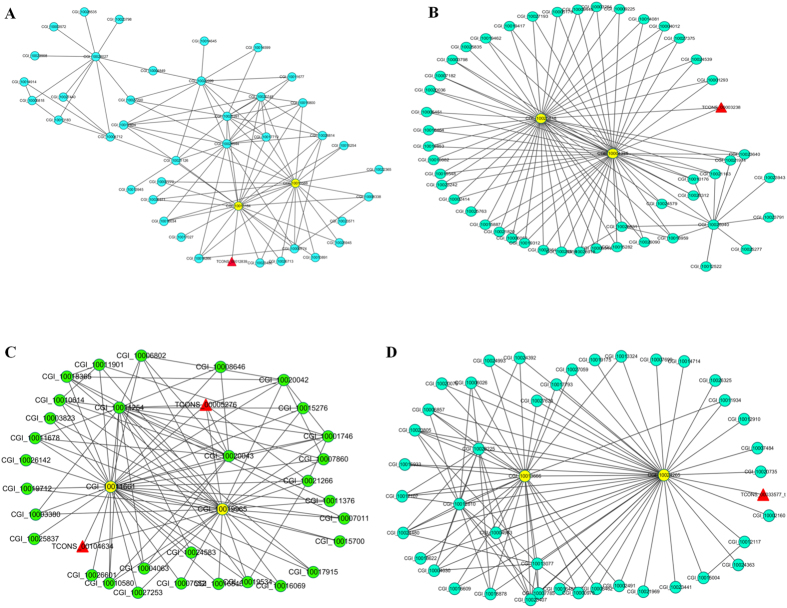
lincRNA-mRNA co-expression network. Four modules that are significantly correlated with traits were shown. (**A**) Black module. (**B**) Brown module. (**C**) Cyan module. (**D**) Green module. The triangular and round nodes represent lincRNAs and mRNAs, respectively.

Cytoscape analysis of the network was used to identify the hub genes and the putative function of lincRNAs during larval development of *C. gigas*. We defined the 1% (or 5%) of nodes with the highest intramodular connectivity as hub genes, according to the literature^31^. No lincRNA was detected as hub genes in the five modules, but we found five lincRNAs (TCONS_00012839, TCONS_00003238, TCONS_00104634, TCONS_00005276, and TCONS_00033577_t) were directly connected with the key hub genes in four modules (except for turquoise, Fig. 4), indicating that these lincRNAs had expressional correlation with the hub genes and the function might be predicted according to the hub genes. However, the function of the five hub genes connected with lincRNAs in the four modules are yet unknown in *C. gigas*, which would be good candidates for further study. Notably, RING finger protein nhl-1 (CGI_10019965) annotated with GO term “metal ion binding” was identified as a key hub gene during oyster larval development (Fig. 4C). The GO term “metal ion binding” was significantly enriched during oyster larval development. In other bivalves, genes implicated in binding and particularly in ion binding were speculated to be related to the shell biomineralization processes^32^,^33^. Hence, we speculated that the lincRNAs (TCONS_00104634 and TCONS_00005276) might have a potential role in oyster larval shell biomineralization. Unfortunately, due to the lack of the distinct gene function profile for the key hub genes identified in the co-expression network, it hampers the better understanding of how the five interesting lincRNAs act during oyster larval development. Despite this, the present results provide a rich resource for further studies to explore the functional roles of lincRNAs in larval development and molecular mechanisms in oyster larval development.

In summary, our study provides the first catalog of lincRNAs in marine invertebrates, and an insight into the characteristics of lincRNAs in the Pacific oyster. The identified lincRNAs share some common characteristics with vertebrates, such as relatively short length, low exon numbers, low expression, low sequence conservation, and tissue-specific expression. The dynamic pattern of temporal expression of lincRNAs during different developmental stages imply that lincRNAs might have diverse roles in oyster development. The lincRNA-mRNA co-expression network analysis revealed that five lincRNAs might function in larval development by interacting with key hub protein-coding genes. The identification of lincRNAs in *C. gigas* unlocks the toolbox of molluscan molecular genetics for studies on the lincRNA function.

## Methods

The oyster genome and the annotation file were downloaded from the GigaScience database (ftp://climb.genomics.cn/pub/10.5524/100001_101000/100030/genome_v9/and ftp://climb.genomics.cn/pub/10.5524/100001_101000/100030/gene_v9/). Illumina RNA-Seq data were also downloaded from the GigaScience database (ftp://climb.genomics.cn/pub/10.5524/100001_101000/100030/RNA-Seq/), including ten RNA-Seq datasets (paired-end reads, GEO accession number GSM768396-GSM768404, GSM973195) mainly from nine tissues, and 35 different development time-point datasets (single-end reads, GEO accession number GSM768406-GSM768414, GSM768416-GSM768428, GSM768430-GSM768433, GSM768435-GSM768443).

The pipeline employed for lincRNA identification was as follows: (1) RNA-Seq reads were mapped to the *C. gigas* genome (assembly oyster_v9) using TopHat^34^. (2) Aligned reads for each sample were assembled using Cufflinks, and then we obtained the intergenic transcripts for each sample assembly using Cuffcompare^35^. Transcripts with short lengths (<200 bp) were excluded for further analysis, because putative long noncoding RNAs were arbitrarily defined as transcripts that are ≥200 bp^1^,^2^,^6^,^7^,^8^,^9^,^10^,^11^,^13^,^14^,^15^,^16^,^17^. (3) The tool Coding-Non-Coding Index (CNCI) was utilized to detect putative protein encoding transcripts which were discarded for further analysis^36^. (4) Coding Potential Calculator (CPC) was used to assess the protein-coding potential of a transcript again, and the transcripts with protein-coding-score larger than zero were eliminated^37^. (5) The transcripts with long ORFs (≥100) were filtered out using Ugene^38^. (6) To rule out housekeeping lncRNA (including tRNAs, snRNAs, miRNA and snoRNAs), the transcripts were aligned against the housekeeping lncRNA databases using BLASTN (E-value ≤ 1e-5). The housekeeping lncRNA databases contained the tRNA database downloaded from Genomic tRNA Database (http://gtrnadb.ucsc.edu/download.html); miRNA database from miRBase (http://www.mirbase.org/ftp.shtml); ribosomal RNA database from SILVA database (http://www.arb-silva.de/download/arb-files/). (7) Transcripts located within the 500 bp flanking regions of annotated protein-coding genes were removed considering that these sequences might be extended exons of annotated protein-coding genes^8^,^16^,^24^. (8) The tool CD-HIT was used to cluster the putative lincRNAs with an identity of 0.95, and the longest sequence in the cluster was selected for further analysis^39^.

The lincRNA homologs in four molluscan genomes (*L. gigantea, A. californica, B. glabrata* and *P. fucata*) were investigated. The genomes of the four species were downloaded from the NCBI genome database (ftp://ftp.ncbi.nlm.nih.gov/genomes/), MarinegenomicsDB (http://marinegenomics.oist.jp/genomes/gallery), Ensembl Metazoa (http://metazoa.ensembl.org/index.html), and VectorBase (https://www.vectorbase.org/organisms/biomphalaria-glabrata), respectively. The lincRNA sequences identified in *C. gigas* were aligned against the four molluscan genomes with BLASTN (E-value ≤ 1e-5, sequence identity >20%).

RSEM^40^ was used for expression analysis of lincRNAs and mRNA to obtain FPKM values. HTSeq-count^41^ was used to obtain raw counts of reads. We used the tissue-specific index to evaluate tissue-specific expression of lincRNAs. The index was calculated as described by Hao *et al.*^16^. The expression value of each lincRNA was quantified as FPKM. The lincRNAs with tissue-specific index >0.95 were considered as tissue-specific lincRNAs. The heat map of lincRNA expression in different tissues was constructed using heatmap.2 function of R packages (version 3.2.0).

RNA-Seq data of 34 different larval development time-points (from eggs to spats) were used to construct a lincRNA-mRNA co-expression network. First, we calculated the expression of lincRNAs and mRNA in each development time (FPKM). Weighted gene co-expression network analysis (WGCNA)^42^ was used to describe the correlation patterns among mRNAs and lincRNAs coding genes across the 34 samples, from which, we found modules of highly correlated genes by WGCNA R library^43^. We also evaluated the correlation between modules and sample traits which included three trait datasets: actual development time (day), “time order” and development stages (Table S1). Cytoscape was employed for visualization of the co-expression network^44^. The mRNA genes in the larval development trait-related modules were analyzed by Gene Ontology (GO). GO terms enrichment analysis was performed using the hypergeometric distribution and Bonferroni correction for multiple hypotheses testing.

## Additional Information

**How to cite this article**: Yu, H. *et al.* Genome-wide identification and characterization of long intergenic noncoding RNAs and their potential association with larval development in the Pacific oyster. *Sci. Rep.* **6**, 20796; doi: 10.1038/srep20796 (2016).

## Supplementary Material

Supplementary Information
